# Tracking and Mining the COVID-19 Research Literature

**DOI:** 10.3389/frma.2020.594060

**Published:** 2020-11-06

**Authors:** Alan L. Porter, Yi Zhang, Ying Huang, Mengjia Wu

**Affiliations:** ^1^Search Technology, Inc., Norcross, GA, United States; ^2^Science, Technology & Innovation Policy, Georgia Tech, Atlanta, GA, United States; ^3^Faculty of Engineering and Information Technology, Australian Artificial Intelligence Institute, University of Technology Sydney, Ultimo, NSW, Australia; ^4^Department of Management, Strategy and Innovation (MSI), Center for R&D Monitoring (ECOOM), KU Leuven, Leuven, Belgium; ^5^School of Information Management, Wuhan University, Wuhan, China

**Keywords:** text analysis, tech mining, bibliometrics, COVID-19, coronavirus, pandemic

## Abstract

The unprecedented, explosive growth of the COVID-19 domain presents challenges to researchers to keep up with research knowledge within the domain. This article profiles this research to help make that knowledge more accessible via overviews and novel categorizations. We provide websites offering means for researchers to probe more deeply to address specific questions. We further probe and reassemble COVID-19 topical content to address research issues concerning topical evolution and emphases on tactical vs. strategic approaches to mitigate this pandemic and reduce future viral threats. Data suggest that heightened attention to strategic, immunological factors is warranted. Connecting with and transferring in research knowledge from outside the COVID-19 domain demand a viable COVID-19 knowledge model. This study provides complementary topical categorizations to facilitate such modeling to inform future Literature-Based Discovery endeavors.

## Introduction

Never before has there been an explosion of research literature like that taking place this year on COVID-19 [COVID-19 stands for COrona VIrus Disease, 2019, due to the (novel) SARS-CoV-2 virus strain]. This rich literature attracts bibliometric community attention to develop data-driven methods and discover insightful knowledge for specific research questions, e.g., investigating the reaction of scientific communities to the COVID-19 crisis (Zhang L. et al., 2020), identifying the patterns of international collaboration (Fry et al., 2020), and profiling COVID-19–related research topics from a scientometric perspective (Colavizza et al., 2020).

We treat a core COVID-19/coronavirus research collection from 1949 through July 1, 2020. With US National Science Foundation (NSF) support (see Funding), we will update biweekly into 2021 to monitor shifting research emphases. Thus, we gain a prior position to mine this rich information resource to (1) profile the explosively growing research and communicate concerning it so as to make findings more accessible to researchers and research managers and (2) pursue Literature-Based Discovery (LBD) techniques to help link research findings to COVID-19 concerns.

We address challenges that entail exploring the dynamics of this remarkable research domain, with specific questions:

➢ Does COVID-19–related research constitute one or more research communities?
➢ How are the players connected? What collaboration patterns over disciplines and countries determine research knowledge transfer (or lack thereof)?
➢ How do research topics congregate? How are research threads connecting or separating in the rapid evolution of this domain?

Section Results Part 1: COVID-19 Research Profile categorizes the research profiling in terms of basic “4W” questions for this research landscape: Who? Where? When? What? We then analyze the research to probe topical emphases and relationships. We examine which topics show accelerating attention in the core research literature over recent months. We then focus on extracting empirical evidence on which of a set of factors pertaining to pandemic resolution are being pursued in the core COVID-19 literature.

As noted, we aspire to uncover useful research knowledge by gaining perspective on the research patterns and helping topical experts identify and associate research threads. We use an approach of scientific evolutionary pathways (SEPs) (Zhang et al., 2017) to identify research topics and communities and track their predecessor-descendant relationships, for the core literature, covering the time period from 1949 to July 1, 2020.

We observe eight major research threads comprising the technical backbone of coronavirus research. COVID-19 connects with influenza and the two pandemics—SARS, 2003, and MERS, 2013. The rising concerns of the public on public health and epidemiology (e.g., quarantine, early detection, and susceptible populations) can be traced during the COVID-19 crisis. However, we also notice a low semantic similarity between topics generated in 2020 and those of previous research, which might indicate novel clinical characteristics of COVID-19 and insufficient knowledge resident in research communities on handling this crisis to date.

We lastly suggest future research challenges regarding this speeding domain.

## Background

We blend bibliometric (c.f., De Bellis, 2009) and text mining methods (c.f., Porter and Cunningham, 2005; Glänzel et al., 2019; Ranaei et al., 2019) to profile research literature (Porter et al., 2002; Zhou et al., 2014).

The bibliometric community has tracked the development and trajectory of many Science, Technology & Innovation (ST&I) domains, including emerging domains (c.f., Guan and Liu, 2014; Glänzel et al., 2019). Our research group has contributed actively to analyses of emerging technologies. In this article, we apply our Tech Emergence Indicator (Carley et al., 2018; Porter et al., 2018) to COVID-19. We have analyzed various emerging ST&I domains, including nanotechnology subtopics, such as solid lipid nanoparticles (Zhou et al., 2019), nano energy (Li et al., 2017), and nano-enabled drug delivery (Ma et al., 2016); solar cells via advanced text analysis (Wang et al., 2015); big data (Zhang et al., 2016, 2019); 3D printing (Huang et al., 2017); and synthetic biology (Shapira et al., 2017). None of those fields have shown growth anything like that of COVID-19 research. COVID-19 is a different focus than an emerging technology—addressing different aspects relating to a viral infection pandemic. Moreover, its research literature is exploding.

Our interests include mapping the leading players and their interconnections, as these evolve over time. Such networking analyses can help understand the research community and detect possible gaps (e.g., opportunities to connect players and topics that could be synergistic) (Huang et al., 2019). It can be fruitful, as well, to explore connections across fields and the involvement of “border fields” (Porter et al., 2019). Transfer of research knowledge to clinical practice poses many challenges (c.f., Ma et al., 2019); COVID-19 confronts special such challenges as a global pandemic.

As noted, our research aspires to identify COVID-19 opportunities for LBD efforts to follow. We want to elicit coronavirus attributes and contributing factors, related biomechanisms and biomarkers, and treatment modalities.

Pioneering LBD development traces to Swanson (1986) discovery of a potential treatment for constriction of smaller arteries in the extremities (Raynaud disease) hitherto not considered in the literature on the disease. By finding common elements, Swanson was able to bridge separated research knowledge, opening up new opportunities. Gordon and Lindsay (1996) extended Swanson's explorations on Raynaud disease. Swanson and Smalheiser (1997) developed the LBD analytical processes. Smalheiser and colleagues (Smalheiser and Swanson, 1998; Smalheiser et al., 2009) made the analytical process much more accessible via software – tools Arrowsmith.^1^

LBD seeks to infer novel, credible, and informative knowledge by explicitly or implicitly associating two or more disparate literature concepts (Bruza and Weeber, 2008; Ittipanuvat et al., 2014). Its benefits have been pursued by bibliometric researchers and applied for a variety of practical issues, especially in biomedical domains, such as the exploration of potential treatments or gene candidates for diseases (Kostoff et al., 2018; Wu et al., 2020) and uncovering new therapeutic uses for existing drugs (Ding et al., 2013). However, despite high potentials in interdisciplinary research (Small, 2010), LBD still entails a set of problem-driven and/or domain-specific approaches. From a bibliometric perspective, the core methodologies of LBD have been recognized as co-occurrence analysis, which quantifies concepts and relationships with the intention of knowledge inference, such as co-word and co-citation statistics (Lindsay and Gordon, 1999). Those core methodologies are further enhanced by incorporating network analytics, such as heterogeneous network analytics (Sebastian et al., 2017) and link prediction (Kastrin et al., 2016). Beyond that, novel techniques including neural network embedding (Crichton et al., 2018; Wu et al., 2020) and recommender systems (Lever et al., 2018) also stand at the forefront of the LBD trend but have yet not formed a mature research paradigm. Apart from those existing methodologies, domain knowledge becomes necessary for constructing references, taxonomies, and knowledge bases.

Inspired by these pioneer studies, our pilot study simulates literature in a bibliometric stream and develops an approach of streaming data analytics to identify and visualize topics and their relationships over time as evolutionary pathways (Zhang et al., 2017). These efforts provide an adaptable tool of *intelligent bibliometrics* for conducting LBD in a broad range of technological areas and research domains, with limited expert support (Zhang Y. et al., 2020). Those form the main techniques exploited in the analysis of topical evolution in this article to identify further LBD opportunities regarding COVID-19 and beyond (to help mitigate future threats).

## Materials and Methods

The dramatic growth of COVID-19 research literature—refereed journal articles, preprints, etc.—has been accompanied by the emergence of multiple information resources. Websites compiling related research information include the following:

➢ from the Centers for Disease Control: https://www.coronavirus.gov
➢ from the National Institutes of Health: https://www.nih.gov/coronavirus
➢ from the National Center for Biotechnology Information: https://www.ncbi.nlm.nih.gov/sars-cov-2/
➢ the consortial CORD-19 (COVID-19 Open Research Dataset), including query (same standard COVID-19 core with related coronavirus research, provided by NLM, that we use) results from the PubMed Central database (an expanded version of PubMed); bioRxiv and medRxiv preprints; and World Health Organization (WHO) articles—compiled by a partnership including Allen Institute for AI (AI2—https://scisight.apps.allenai.org), the Chan Zuckerberg Initiative, Georgetown University's Center for Security and Emerging Technology, Microsoft Research (MSR), NLM, the White House, and Unpaywall.^2^ See https://www.kaggle.com/allen-institute-for-ai/CORD-19-research-challenge
➢ from MIT Press, a site including social media content: https://www.mitpressjournals.org/doi/abs/10.1162/qss_a_00066.

Bibliometric and “tech mining” analyses thus have multiple candidate resources with various attributes. Selection entails tradeoffs of coverage, currency, indexing, peer review or not, abstract records or full text, etc. In our NSF project, we intend to complement the use of a core PubMed dataset with clinical trials.gov and WoS. Some variations include the following: Haghani and Bliemer (2020) and Homolak et al. (2020) used Scopus as well; Liu et al. (2020) complemented PubMed with EMBASE; Niehs' group (https://www.collabovid.org) draw upon PubMed, Elsevier, and three preprint resources—medRxiv, bioRxiv, and arXiv. Lima et al. (2020) reviewed the domain drawing upon PubMed, Scopus, Cochrane Library, and Biblioteca Virtual de Saude (BVS).

Our core COVID-19 search is conducted in the PubMed database [https://www.ncbi.nlm.nih.gov/pmc]. The National Library of Medicine (NLM) therein provides compiled abstract records that can be downloaded openly^3^. We apply the standard NLM COVID-19, inclusive of historical research literature, search query:

➢ “COVID-19” OR coronavirus OR “corona virus” OR “2019-nCoV” OR “SARS-CoV” OR “MERS-CoV” OR “severe acute respiratory syndrome” OR “Middle East respiratory syndrome^4^.”

We use the legacy version of the PubMed database [by shifting from “PMC” via pull-down to “PubMed”] on the website.^5^ NLM offers alternative COVID-19–oriented search queries; this one focuses on COVID-19, but also provides relevant historical research literature on coronaviruses, etc.

On September 6, 2020, afternoon, we ran some data comparisons. The NLM search query that we applied links to an NLM site with the standard search query preloaded. This query version is the NLM “coronavirus broadly (historical and current research)” version. It yields the following:

➢ 70,193 in PubMed (legacy) database
  [for 1949–2021; of which 51,734 date 2020 or 2021 (a nominal 27 publications, indicating a certain lack of accuracy in the dating)]
  [contrast that to the 47,607 in our analyses based on PubMed (legacy) search on July 1—up almost 50% in 2 months!]
➢ 97,457 in PMC (PubMed Central)
  [this extends coverage to more preprints and such—more current, but less controlled publication types]
➢ This NLM search site offers a choice of some 30 alternative databases to search. Running the same search query in “All Databases” finds hits in various research literature, gene, protein, genome, and genetics databases.
  [Our focus is on research literature, so the most suitable choices are PMC (wider coverage) or PubMed (better controlled and indexed content).]
➢ The CORD-19 Open Research Dataset (noted above) shows more than 200,000 articles, including about half with full text (not suitable for our present comparative analyses).

We also compared certain published work on COVID-19. For example, Colavizza et al. (2020) focused on the CORD-19 dataset, but facilitated altmetric data (e.g., Twitter and Blogs) to track the dissemination of publications across various social media sources. Fry et al. (2020) conducted their COVID-19 search based on PMC-sourced materials from the CORD-19, as well, but they combined related articles retrieved from WoS and Scopus by the same search strings. Slightly different from the above, Zhang L. et al. (2020) concentrated their study on COVID-19 by a comparison with four selected viruses (e.g., Zika, H1N1, SARS, and Ebola), and they collected their COVID-19 data from WoS, PubMed, and CNKI (Chinese National Knowledge Infrastructure) in the early days of the crisis, with ~3,000 hits.

We post these search results relating to this core COVID search on the website: http://www.techminingforglobalgood.org/open-covid-19-research-for-analysis/, as a *VantagePoint* file. That text analytic software (*VantagePoint*) is available for users to apply to answer their research questions using these data.

The Supplementary Material includes a screenshot (Supplementary Figure 1) of the *VantagePoint* summary sheet describing July 1, 2020, a data file of 47,607 abstract records of publications indexed by PubMed. Those include 36,184 journal articles (76%). Some points of note as the following:

➢ Some 50 additional data fields can be readily imported to augment the fields shown; see screenshot [e.g., “Abstract [Natural Language Processing (NLP)] (Phrases)”].
➢ Various fields can be examined as separate lists or combined as a matrix to explore relationships.
➢ Elements can be extracted from fields (e.g., countries from author affiliations).
➢ Cleaning and consolidation can be performed (e.g., fuzzy matching and stemming from consolidating topical terms, applying thesauri to remove many unhelpful terms).
➢ Groups of items can be formed and used to form new fields (e.g., bunching publication dates into three time periods).

To illustrate “zoom in” capabilities, Supplementary Figure 2 shows a screenshot of a “sub-sub-sub-dataset.” We first separate the 29,156 COVID-19 PubMed records dated 2020. We then apply a thesaurus that separates affiliations into their most likely category of academic, corporate, government, and/or hospital—creating a sub-dataset of the hospitals. We then separate the 699 records with hospital affiliations with a US location. The screenshot spotlights the 43 publications with a Brigham and Women's Hospital author or co-author. The five titles by Meissner are highlighted. The detail windows on the right show high-frequency topics covered by the 43 articles—MeSH Descriptors, terms related to the eight factors pertaining to tactical vs. strategic emphases, and NLP-extracted terms. In essence, the data are thereby made accessible to begin to identify one's interests therein.

**Figure 1:**
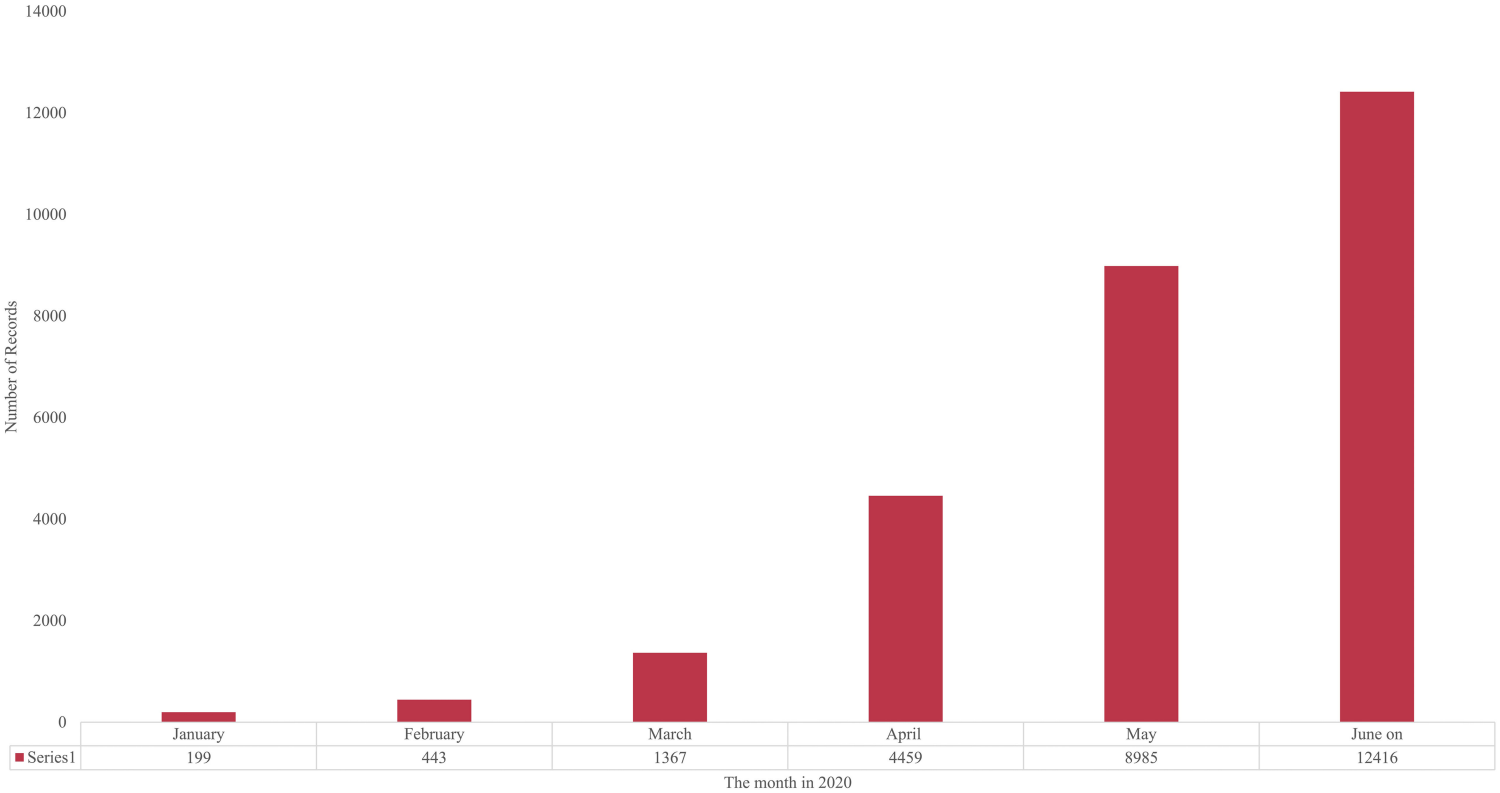
Growth in COVID-19–related Publications Indexed by PubMed, Monthly, January-June, 2020.

**Figure 2:**
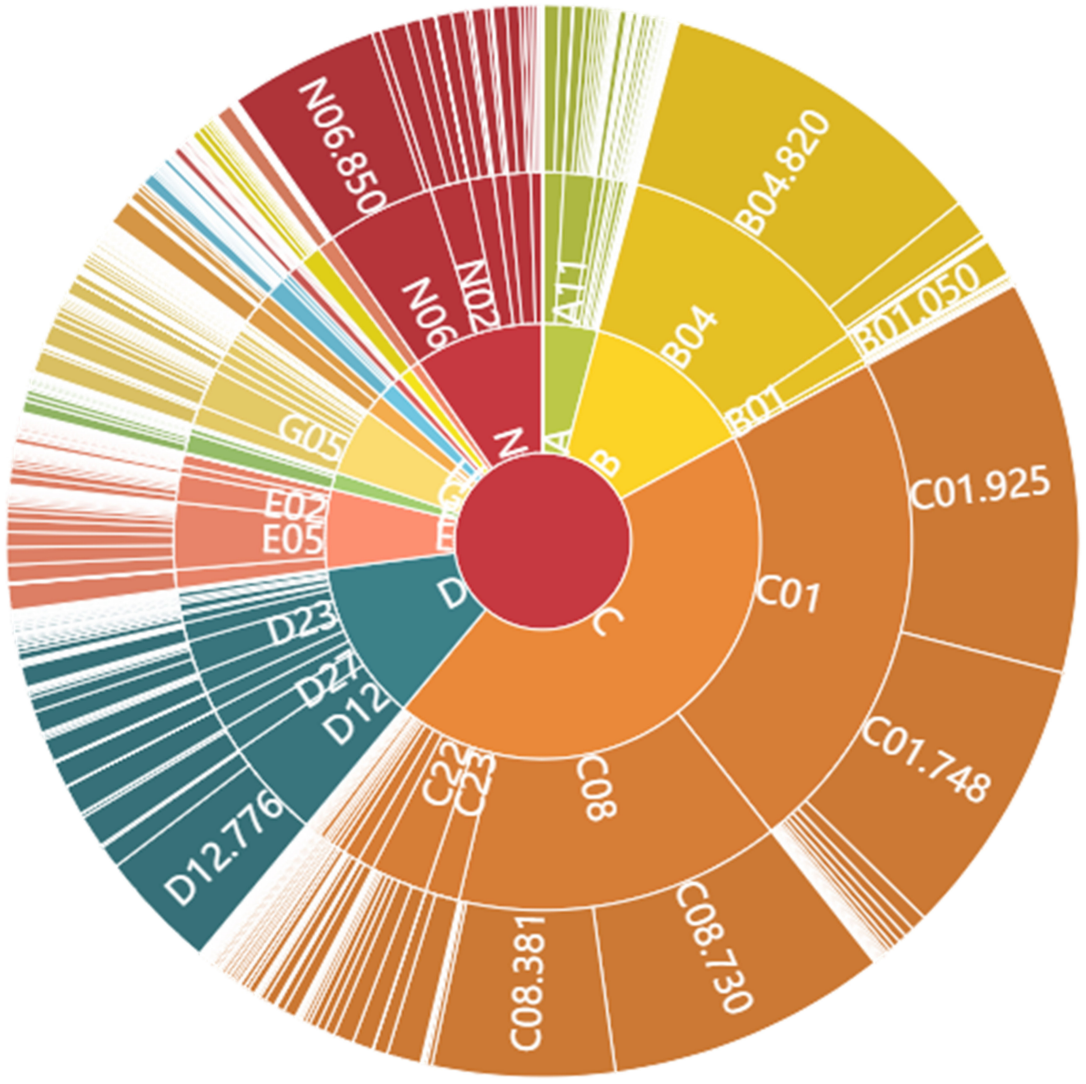
Medical Subject Headings (MeSH term) Distribution Consolidated to 4 Levels.

For our several analyses, we categorize publication dates^6^ in several ways. We do so to focus more suitably on particular analytical inquiries. These include the following^7^:

➢ Tables 1, **4** compare the entire period (47,607 articles) to 2020 publications.
➢ **Figure 4**, as well as Supplementary Figure 3, track topical evolution for the entire period, 1949 through 2020 (part-year).
➢ **Table 3** compares two periods: 1949–2019 (“historical”) vs. 2020 (to date, the search conducted on July 1) to sharpen perspective on topical emergence.
➢ Figures 1, 2 accentuate evolving disciplinary contributions for monthly COVID-19 periods, from January 2020.
➢ **Figures 5**, **6**, like Figure 2, focus on the COVID-19 era of 2020 publications.

**Table 1:** Leading organizations publishing COVID-19–related papers^8^.

**No**.	**Organizations**	**1949–2020** **(47,607)**	**2020** **(29,156)**
1	Univ of Hong Kong	547	157
2	Huazhong Univ of S and T	425	393
3	Chinese Univ of Hong Kong	400	112
4	Chinese Acad of Sciences	311	91
5	Wuhan Univ	298	239
6	Univ of Pennsylvania (2)	268	115
7	Utrecht Univ	241	30
8	Johns Hopkins Univ	240	141
9	Univ of Toronto	220	107
10	Nat Univ of Singapore	214	136
11	Univ College London	211	183
12	Univ of So. Cal.	211	30
13	Fudan Univ	210	129
14	Univ of California	206	50
15	Univ of North Carolina	198	38
16	Chinese Acad of Med Sci	190	120
17	Univ of Iowa	190	34
18	Univ of Washington	182	131
19	Chinese Acad of Agri Sci	181	14
20	Ohio State Univ	164	31
21	NIAID	158	44
22	Ministry of Health, Saudi Arabia	139	38
23	Tan Tock Seng Hospital	114	57
24	Nat Inst of Infectious Diseases, Japan	105	6
25	Univ of Georgia	105	1

**Figure 3:**
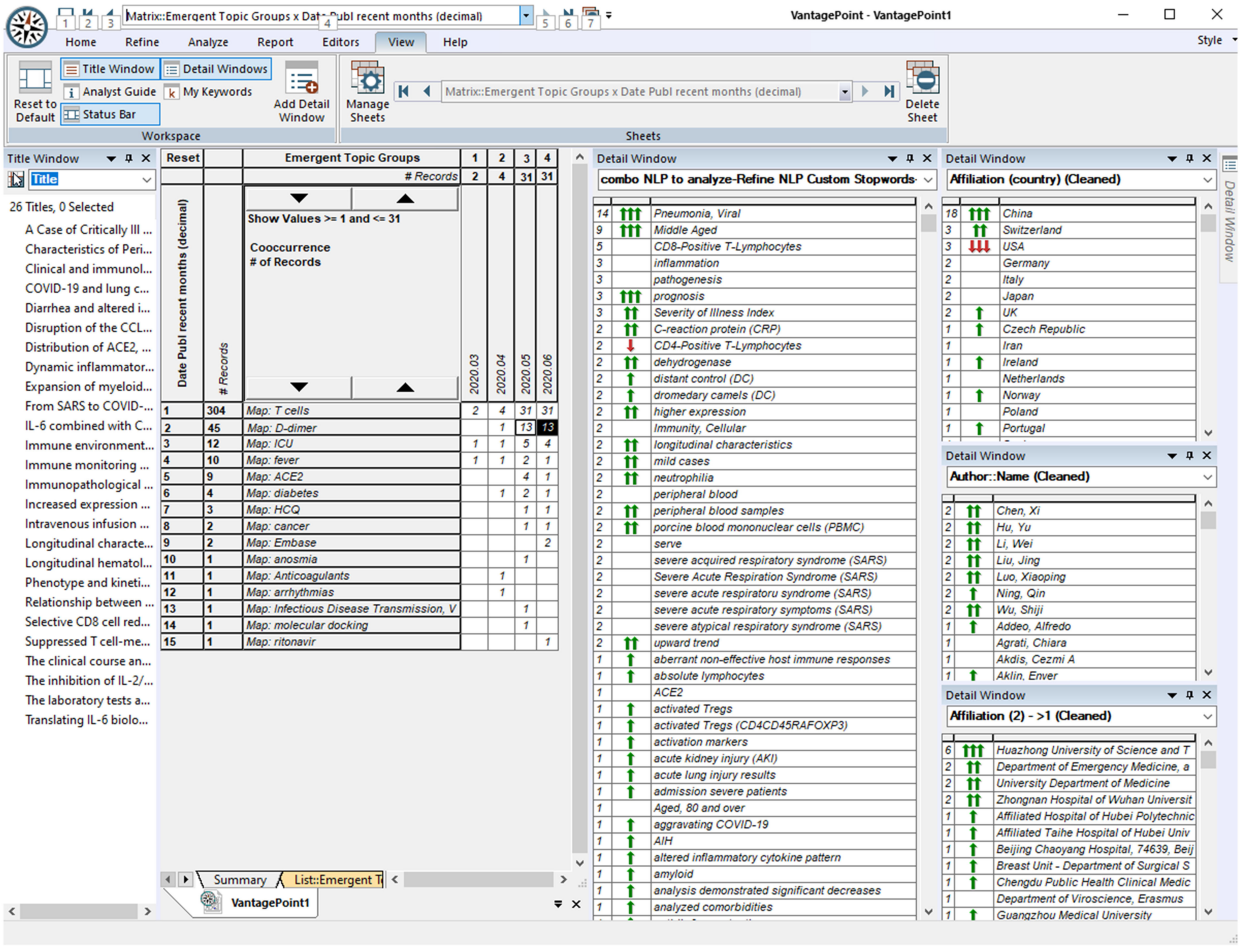
Screenshot of *VantagePoint* Software: Exploring Combinations of Who, Where, When, and What?

Figure 3 and Supplementary Figure 1 offer a glimpse of fields available for various analyses. As we have done biweekly data downloads and analyses starting March 25, 2020, we have refined the raw data various ways. To anticipate the “4W's” of section Results Part 1: COVID-19 Research Profile, we note basic cleaning in *VantagePoint* includes the following:

➢ **Who?** Particularly consolidate author organizational name variations using a combination of COVID-19 dataset affiliation names in a continually growing thesaurus, along with a List-Cleanup fuzzy matching routine tailored to PubMed data formatting^9^ —e.g., to extract from “Department of Radiology, Union Hospital, Tongji Medical College, Huazhong University of Science and Technology, Wuhan, Hubei, China.” “Huazhong Univ of S&T”
➢ **Where?** Author country location extracted from strings such as that just given, leading to building of a COVID-19–oriented thesaurus to clean further (e.g., to ensure “PR of China” appears as China, not Puerto Rico too).
➢ **When?** The challenge posed by COVID-19 data analyses is their unprecedented pace. We are used to handling annual publication dates. However, we sought something finer here. We explored various of the eight or so provided dates—e.g., Entrez Date, Date Created, but deemed Date Published as most effective. While recognizing that monthly dates are not fully accurate reflections of research timing, we use these in analyzing topical emergence. To consolidate at a monthly level, we have built a date thesaurus. For instance, this would tag as “2020.06 (June)” such variations as 2020 Jun, 2020 Jun 1, 2020 Jun 25, 2020 Summer, 2020 Jun/Jul, etc.
➢ **What?** As discussed in section “What?” we address multiple topical resources that pose multiple data quality issues. MeSH terms, as indexed by NLM, are invaluable, but MeSH indexing of recent month publications lags. Ying Huang devised an approach to consolidate related topics across four levels that facilitates topical analyses using MeSH. Abstracts are available for some 65% of the record set. We extract phrases from titles and abstracts using *VantagePoint* NLP that is tailored to scientific discourse^10^^,^^11^. We apply a routine with multiple thesauri (e.g., “scientific & academic stopwords”; “common & basic stopwords”)^12^; and a fuzzy matching List-Cleanup routine to consolidate phrase variants. For certain analyses, we combine these NLP phrases with MeSH Descriptors or Level 3 terms.

## Results Part 1: COVID-19 Research Profile

We first overview the “Who? Where? When? What?” of this explosively growing research literature [e.g., our biweekly updates, using the standard NLM historical and COVID-19 search query have grown from 19,538 publications on March 25, 2020, to 47,607 on July 2 (used in the present analyses), and 70,193 as of September 6]. Haghani and Bliemer (2020) and Thelwall (2020) conducted the case that historical research bears importantly on current COVID-19 research. Such profile information should facilitate identifying and locating key players and their research foci. That intelligence, in turn, can help identify research topics to consider oneself, key sources, and potential collaborators. It may also stimulate more detailed inquiries one might pursue. To set the stage, here are select “4W” basics.

### The 4 W's: Who? Where? When? What?

This section profiles prominent features of the COVID-19 research landscape to gain perspective on research players and trends based on the core PubMed dataset downloaded on July 1, 2020 (47,607 records).

#### Who?

These 47,607 published articles are authored by a strikingly broad array of researchers (150,000+), organizations (after basic cleaning, 5,819 with multiple publications), and countries (191). We treat countries under “Where?” and we leave the individuals for exploration on the website (7,296 with five or more articles in the dataset).

Table 1 lists the Top 25 organizations for this dataset, coinciding with >100 articles^13^. The rightmost column shows how many of those articles appeared with 2020 dates. Note that research emphases shift sharply—e.g., #25, the University of Georgia, has not been very active this year. Conversely, #2, Huazhong University of Science & Technology, which is in Wuhan—the central city of the early COVID-19 pandemic, has been more active this year (only 32 pre-2020 articles vs. 393 so far this year). Such shifts reflect the transition from study over decades of coronaviruses and the SARS and MERS outbreaks, vs. COVID-19 pandemic–motivated studies.

The USA (with 10) and China (8, including Hong Kong) dominate this list of top organizations.

Again, anyone interested in details is invited to visit our websites to explore the dataset.^14^ Information such as the following is available:

➢ Ability to search for a particular organization's or an author's publications in the 47,607 set;
➢ Topical emphases by particular players using Medical Subject Heading (MeSH) Qualifiers or Descriptors, or title and abstract phrases;
➢ Connections among players (e.g., maps or matrices of who collaborates with whom).

#### Where?

The 47,607 articles in the dataset are associated with 196 countries. Of those, 12 appear as author affiliations on >1,000 articles; 45 have >100. Two city–states are in the top 20—Singapore and Hong Kong. The leading two countries are the USA (9,843) and China (7,400). We are interested to see Top 20 contributions from Iran (613) and Saudi Arabia (613) and note Saudi—the initial center of MERS pandemic—interests in MERS have continued actively since 2014.

➢ USA−2,874 of 9,843 articles (29%) show international co-authoring, most with China (729)
➢ China−1479 of 7,400 articles (20%) show international co-authoring, most with the USA (729)
  [China–USA collaboration remains strong in most recent months, despite political forces−13% of 1,550 Chinese articles dating June or beyond are joint with US authors, compared to 8% of Chinese COVID-19–related publications prior to May. This finding coincides with that observed by Fry et al. (2020)—the COVID-19 crisis accelerates the international collaboration in and between China and the USA].
➢ Italy−1,223 of 3,635 articles (34%) show international co-authoring, most with the USA (453)
➢ Extensive European co-authoring among some 20 European countries in the Top 45.

#### When?

The trend in COVID-19–related research is astounding. A plot of the time series from 1949 through 2020 (part-year) appears in the Supplementary Material, see Supplementary Figure 3. Growth is hyper-exponential.

Academia can often respond quickly to public health emergencies. We see a sharp increase in the number of publications immediately following the declaration of an outbreak by the WHO. To estimate the recent COVID-19 research growth, we divide the publication dates into months from September 2019 through “June on,” 2020.^15^ Figure 1 plots the monthly data from January 2020 [For the last 4 months of 2019, the count is mostly flat, ranging from 58 to 78 articles indexed by PubMed]. That more than doubled in January, and again in February, and tripled in March, and again in April. The proportionate rate of growth calmed somewhat, doubling from 4,459 publications in April to 8,985 in May, then increasing somewhat less (by 3,431) to June (on)'s 12,416. However, in perspective, we have gone from fewer than 100 articles/month to more than 12,000 per month in less than a year.

#### What?

A prime reason to compile COVID-19–related research is to discover research knowledge that can be brought to bear in helping to remedy the pandemic and prevent or mitigate future ones. Toward such ends, we aspire to make the topical content accessible and relatable to one's research questions.

The 47,607 abstract records from PubMed offer several *topical* information resources:

➢ Titles (for 99% of the records)
➢ Abstracts (for 65%)
➢ MeSH indexed terms (for 56%), including Qualifiers (76 categories found for this dataset), Descriptors (9,412), and Primary Terms (27,174)

To enhance those, we have several derived topical fields:

➢ *VantagePoint* extracted NLP phrases or words from Titles and Abstracts
➢ Merged fields, especially “Combo NLP” made of Abstract NLP phrases and Title NLP phrases and MeSH Descriptors combined [each field and the combined field having been refined using a combination of stopword thesauri and fuzzy matching routines (“Refine NLP” script in *VantagePoint*)]—to yield 392,247 terms (covering 96% of the records to some extent)
➢ Emergent terms (phrases) evidencing accelerating recent growth (1,380) (how these are calculated is discussed shortly)

We provide select top-level synopses to gain perspective on this topical content. Figure 2 illustrates the complexity of the MeSH hierarchical index terms, showing the top three levels. On the one hand, some related concepts are listed in multiple levels—e.g., “severe acute respiratory syndrome (SARS)” (Unique ID: D045169) is allocated to three sub-fields (Tree Numbers) at two different levels:

➢ C01.748.730 (Level 4) under “respiratory tract infections [C01.748]”
➢ C01.925.782.600.550.200.750 (Level 8) under “coronavirus infections [C01.925.782.600.550.200]”
➢ C08.730.730 under “respiratory tract infections [C08.730]”

On the other hand, an article might be indexed with several MeSH terms—e.g., The article “Localization of mouse hepatitis virus open reading frame 1A derived proteins” (PMID: 10065901) has Primary MeSH terms (no subheading):

➢ DNA, viral ([D13.444.308.568])
➢ Murine hepatitis virus ([B04.450.580] and [B04.820.578.500.540.150.113.875])
➢ Open reading frames ([G05.360.335.760.640], and [G05.360.340.024.340.137.650])
➢ Viral proteins ([D12.776.964])

Therefore, we cannot compare them to tally high-frequency MeSH terms directly. So, we set out to consolidate the lower level items to the upper level. When we want to analyze them on Level 4, the Tree Number of the article mentioned above can be presented as D13.444.308, B04.450.580, B04.820.578, G05.360.335, G05.360.340, and D12.776.964. So, we determined to consolidate related terms at different levels by using a thesaurus to group related MeSH terms.

In terms of the MeSH hierarchy, we can follow the tree structure up or down. For example, going downward, Level 1 (single letter) includes diseases [C]. Under that at Level 2 (letter–number), one category is infections [C01]. Under Infections, at Level 3 is virus diseases [C01.925]. Under virus diseases at Level 4 is RNA virus infections [C01.925.782]. Coverage can be impressively extensive. For instance, in this core COVID-19 dataset, more than half the records (24,796) are associated with that Level 4 topic—RNA virus infections.

From Figure 2, we see that the virus diseases [C01.925] in infections [C01], and RNA viruses [B04.820] in viruses [B04] are the most common MeSH topics (sub-fields) in this COVID-19 dataset. Those are followed by respiratory tract diseases [C01.748, C08.730], a converged sub-field in the above two fields. Besides, lung diseases [C08.381], public health [N06.850], and proteins [D12.776] are three other notable sub-fields in COVID-19 research. Overall, the diseases [C] cover the most records, followed by organisms [B], chemicals and drugs [D], and healthcare [N] in Level 1. The Top Primary MeSH terms in Levels 2 to 4 for these COVID-19 core publications set are shown in Supplementary Tables 1–3.

We explore “emerging topics” —a particular subset of topical information of high potential value—in section Results Part 2: Special Analyses.

### 4W's in Combination

Combinations of the Who, Where, When, and What factors offer insights into research concentrations. Select comparisons have appeared in section The 4 W's: Who? Where? When? What?—e.g., emergent topics shifting over time. We present a few combinations here and invite exploration of one's particular interests on the website.

Table 2 breaks out topics using the top 16 MeSH Qualifiers (>2,000 associated records) by the top countries, showing the percentage of a country's articles that address the given topic (for all years in the dataset). Research policymakers or managers might be interested in relative emphases, as these topics correspond somewhat to disciplines. For instance, Japan shows the highest concentration on virology (32.5%), with India the least (7.6%). Genetics and immunology are less emphasized in India, Italy, and the UK, but more in the Netherlands and Japan. Or, one could scan by country, noting that Japan's emphases seem somewhat different on multiple categories (e.g., the highest percentage on some nine of these domains). Or one might compare particular countries—the USA and China appear to hold generally similar research interests regarding COVID-19. Similar breakouts of topical categories by organization or author enable detailed exploring for expertise focused on one's intersection of interests.

**Table 2:** COVID-19 topics (top 16 MeSH Qualifiers) by country (top 12), for all years (47,607 records).

	**Records**	**9,843**	**7,400**	**3,635**	**3,548**	**1,851**	**1,716**	**1,538**	**1,416**	**1,159**	**1,134**	**1,096**	**1,044**
**Records**	**MeSH qualifiers**	**USA**	**China**	**Italy**	**UK**	**Canada**	**France**	**Germany**	**India**	**Australia**	**Spain**	**The Netherlands**	**Japan**
8,704	Epidemiology	13.9%	19.1%	14.2%	17.9%	20.5%	16.6%	12.8%	14.0%	19.8%	10.6%	13.7%	14.8%
8,051	Virology	20.5%	22.5%	10.3%	11.3%	14.5%	14.1%	20.6%	7.6%	14.7%	10.0%	25.9%	32.5%
6,821	Genetics	16.4%	20.2%	5.2%	8.3%	13.0%	10.7%	18.8%	4.6%	9.8%	12.9%	29.9%	25.6%
5,252	Immunology	12.2%	13.2%	4.3%	5.6%	11.2%	7.1%	10.7%	2.6%	6.0%	9.3%	14.5%	21.1%
5,058	Isolation and purification	8.7%	12.7%	6.2%	6.3%	9.5%	8.6%	10.2%	4.2%	8.6%	4.8%	11.1%	15.8%
5,054	Prevention and control	10.2%	9.9%	6.6%	10.5%	13.8%	7.9%	7.9%	7.9%	12.3%	6.0%	9.8%	9.1%
4,106	Metabolism	11.9%	11.7%	2.5%	4.7%	8.4%	6.8%	16.7%	3.5%	2.6%	8.1%	21.4%	16.2%
3,981	Diagnosis	6.1%	11.6%	6.5%	6.7%	6.9%	7.8%	8.3%	4.6%	6.5%	4.8%	6.0%	8.0%
3,734	Methods	7.8%	9.4%	5.8%	7.2%	9.6%	6.0%	7.6%	5.9%	8.4%	5.5%	8.2%	8.7%
3,385	Veterinary	6.9%	6.4%	3.9%	4.3%	4.4%	4.0%	4.8%	1.4%	6.0%	3.4%	7.8%	16.2%
3,074	Physiology	9.9%	6.3%	1.9%	3.7%	7.1%	5.8%	10.2%	2.7%	3.5%	6.7%	16.9%	14.2%
2,829	Transmission	5.1%	7.0%	4.0%	6.1%	6.4%	5.2%	5.1%	3.5%	6.9%	2.8%	5.9%	4.0%
2,481	Pathogenicity	6.7%	6.1%	2.6%	2.6%	4.9%	3.1%	6.6%	2.5%	3.4%	4.7%	7.9%	10.9%
2,377	Chemistry	7.1%	7.4%	1.4%	2.4%	4.2%	4.1%	9.0%	2.5%	1.6%	5.7%	9.2%	9.4%
2,341	Therapy	4.5%	6.6%	6.5%	5.4%	5.7%	6.2%	5.1%	3.7%	5.6%	4.6%	3.1%	2.1%
2,156	Pathology	5.6%	5.8%	2.3%	1.7%	5.2%	2.8%	3.8%	2.2%	2.6%	1.9%	5.1%	8.8%

Crossing topics by topics facilitates the identification of important confluences. This is likely to yield valuable intelligence at quite specific levels. A matrix of the 29 emergent topics by the 29 emergent topics could provide useful leads. To illustrate, within this dataset of COVID-19–related articles, someone pursuing immune system attributes might be especially interested in the seven articles considering T cells and immunoglobulin M.

Three (or more) dimensional combinations can also be highly informative. We do not attempt to show many of those here, but the website enables tailored exploration. Just to illustrate, we separate the 304 of the 47,607 COVID-19–related records concerning T cells. Then, we select the subset of 45 records, also considering d-dimer (relevant to diagnostic testing for blood clotting). The screenshot (Figure 3) shows the selection of the 26 of those published since May 2020. The window on the left gives the article titles. The detail windows on the right show the following:

➢ This research is concentrated in China, especially at Huazhong University of Science and Technology, with many authors (no high concentration in one person who might be the laboratory leader).
➢ It relates mainly to pneumonia, especially with middle-aged persons.

Probing a little deeper, can we tell who might be key researchers on the topic at Huazhong? The six articles show 137 authors. Mapping just those co-authoring two of the articles, we see two groups: Li and Hu, and Wu, Ning, and Luo. So, if we want to pursue, we might read the abstract records of each and possibly do further research by finding the full articles to read.

## Results Part 2: Special Analyses

### Emerging Topics in COVID-19 Research

We score terms appearing in the titles and abstracts on the extent to which they are “emergent” —i.e., terms evidencing increasing attention in most recent periods (Carley et al., 2018; Porter et al., 2018). This notion of emergence builds upon the research of the US Intelligence Advanced Research Projects Activity (IARPA) Foresight and Understanding from Scientific Exposition (FUSE) Program.^16^ Our contributions to IARPA FUSE conceptualization appear in Alexander et al. (2012). FUSE explored full text datasets and entire databases. Our adaptation is to devise emergence indicators using targeted datasets (e.g., on a particular scientific or technological domain), using abstract record sets (Carley et al., 2018; Porter et al., 2018; US Patent Application 15/803,185).

We draw heavily upon Rotolo et al. (2015) who digest extensive conceptual contributions and empirical research bearing on technical emergence. We set thresholds on four factors:

➢ *novelty* [appearing in <15% of base period (first three periods—here, those are September, October, and November 2019) records],
➢ *persistence* [term appears in at least seven records, in at least three periods],
➢ *community* [at least two organizations using the term in the corpus],
➢ *growth* [term usage grows at least 1.5 times the overall publication growth rate in the dataset being analyzed].

Porter et al. (2018) provide a series of empirical studies that, along with additional studies, contribute to these thresholds for the indicators. By and large, these are based on empirical judgments based on a moderate number of case analyses and consensual opinions by the developers, not strong theoretical foundations. For novelty, allowing a small number of occurrences of a topical term in the base period, rather than zero, retains topics that take a few periods for uptake. Persistence thresholds seek to sidestep one-shot wonders. For community, we seek to avoid singular topics by requiring authorship from more than a single organization. Scope entails consideration of term Inverse Document Frequency (IDF) values to prescreen terms less domain-specific. The growth threshold seeks to distinguish emerging topics in fast-growing domains—especially pertinent to COVID-19–related research. Calculating the tech emergence indicators is facilitated via a *VantagePoint* routine. That macro offers one the opportunity to vary these parameters. One can apply additional ones as well; notably, “scope” can be narrowed by using IDF to screen out terms very common in the dataset.

The script then derives an Emergence Score for each term by weighing three growth rate measures—active trend (change in publication rate from periods 4–6 to 8–10), recent trend (change from periods 7–8 to 9–10), and slope (change in rate from period 7–10). This formulation derives from the oft-observed “S-shaped” growth of emerging technologies. Assessment of the tech emergence indicators suggests interesting potential to help anticipate future research directions and innovation potential (Kwon et al., 2019; Porter et al., 2020). The hyper-exponential growth of COVID-19 research poses a different model (grounds for future research).

Emergence Scoring has been developed using years to constitute 10 time periods. Our algorithm divides those into the first three as a base period and the last seven as the active (growth) period (Porter et al., 2018). We have adopted this model for particularly fast-growing domains by trying shorter periods—especially using half-year research publication or patent filing periods. COVID-19 challenges this emergence model because of its hyper-growth in 2020 (Figure 1 and Supplementary Figure 3). So, to operationalize recent periods, we made 10 groups of records based on publication month, dating from September 2019, through June (on), 2020 (see Figure 1 for the 2020 months). Still, the extreme variation therein (from about 80 articles per month to over 12,000 per month) represents a new venue in which to apply our Emergence Scoring method.

We ran *VantagePoint*'s Emergence Scoring script on the “combo NLP” field of 392,247 terms to spotlight 1,380 Emergent Terms^17^. We then clustered those terms based on their tendency to co-occur in records of the dataset, using *VantagePoint*'s “factor mapping” script that applies a tailored version of principal components analysis (PCA) (Wang et al., 2019).

Table 3 lists the resulting emerging topics and the number of records in which their high loading terms (that constitute each topical cluster) appear.^18^ The PCA routine names the factor for its highest loading term. We edit some of those names—e.g., “patient care teaming,” rather than “pharmacy service, hospital.” That factor has four additional high-loading terms (infection control, intensive care units, health services needs and demand, and interdisciplinary communication); we feel “patient care teaming” better characterized the group of terms.

**Table 3:** COVID-19–related research emergent topics.

**Emergent topic groups**	**Records**	**1949–2019**	**2020 (through July 1)**
Patient care teaming	1464	3.3%	2.9%
Intensive care unit	1414	1.6%	3.9%
Personal protective equipment	1409	2.2%	3.5%
Symptoms (e.g., fever)	1364	2.2%	3.3%
Computed tomography	984	0.7%	2.9%
C-reactive protein (e.g., d-dimer)	820	1.1%	2.5%
Angiotensin-converting enzyme 2	950	1.3%	2.0%
Diabetes	797	0.4%	2.5%
Hydroxycholoroquine	736	0.2%	2.4%
Surveys and questionnaires (health knowledge, attitudes, practice)	663	1.8%	1.2%
Cancer	630	0.4%	1.9%
EMBASE	628	0.4%	1.9%
Mental health	535	0.3%	1.7%
Study protocol (e.g., randomization)	532	0.9%	1.2%
Healthcare disparities (inequities)	492	0.2%	1.5%
Surgery	357	0.1%	1.2%
Anticoagulants (thrombosis)	352	0.1%	1.1%
Ritonavir/lopinavir	319	0.2%	1.0%
Infectious disease transmission, vertical (pregnancy)	308	0.5%	0.8%
T cells	304	1.3%	0.2%
Antibodies (IgM, IgG)	303	1.0%	0.4%
Arrhythmias	259	0.1%	0.8%
Education, medical	220	0.1%	0.7%
Olfactory disorders (anosmia)	212	0.0%	0.7%
Neurological manifestations	212	0.2%	0.6%
Molecular docking	183	0.3%	0.4%
Head and neck neoplasms	176	0.1%	0.6%
Orthopedics	148	0.0%	0.5%
Ophthalmology	112	0.0%	0.4%

These topics spotlight current research priorities to stimulate consideration of one's research emphases as a researcher or research manager. The topics offer what we believe is an effective granularity (vs. the more detailed 1,380 emergent terms or the overall categorizations of Supplementary Tables 1–3).

The right two columns of Table 3 display the percentage of records in the period before 2020, and those in 2020 as of July 1, associated with each emergent (“hot topic”) group. The most prevalent, “patient care teaming,” holds pretty steady, from 3.3% of the pre-2020 articles that include at least one of its six high loading terms, to 2.9% that do so in 2020 articles. A number of the topics show the strong escalation of interest in 2020, including the following whose percentages at least tripled from the pre-2020 historical period to the 2020 COVID-19 era:

➢ medical issues other than respiratory system (olfactory disorders, ophthalmology, surgery, head and neck neoplasms, orthopedics, arrhythmias, anticoagulants, diabetes, mental health, cancer, neurological manifestations)
➢ antivirals (hydroxychloroquine, ritonavir/lopinavir)
➢ computed tomography (CT)
➢ socioeconomic facets (healthcare disparities, medical education)

Some topics show considerably less attention as a percentage of articles in 2020 compared to pre-2020^19^:

➢ social facets (surveys and questionnaires)
➢ immune system facets (antibodies, T cells)

Were one inclined to pursue research on one or more of these emergent topics, the records associated with a given topic (e.g., the 112 addressing an aspect of Ophthalmology regarding COVID-19) could be separated for further analyses (e.g., identifying who is authoring and what related topics they are considering). We are working to develop the noted website^20^ to enable users to do such “zooming in” without needing the software.

### Tactical vs. Strategic Topic Focus: Empirical Exploration of COVID-19–Related Research

Ron Kostoff and colleagues make a vital case: The COVID-19 pandemic is not merely caused by a particular virus (SARS-CoV-2) operating in a vacuum; rather, the disease's consequences reflect how individuals' immune systems interact with that virus (Kostoff, 2020). To a striking degree, certain demographic groups (the elderly and those with weakened immune systems) are highly vulnerable, whereas others (healthy younger cohorts) are nearly invulnerable. That calls into question which societal policies and approaches would best counter the pandemic (Kostoff et al., 2020a,b).

Here, we explore how some terms associated with particular approaches show forth in the COVID-19 research literature. Following Kostoff (2020), we distinguish two types of approaches: tactical (virological—focused on stopping the target virus now) vs. strategic (toxicological—focused especially on strengthening immune systems), along with categories of actions pertaining to each:

➢ tactical (virological orientation)
  ° restricting exposure
  ° antiviral medications
  ° vaccines

➢ strategic (toxicological orientation)
  ° lifestyle factors
  ° iatrogenic factors (side effects of medical treatments—e.g., immunosuppressive drugs, by definition, increase vulnerability to infection)
  ° biotoxins
  ° occupational/environmental factors
  ° psychological/social/economic factors

We searched for some 125 terms (a few with contingencies—e.g., chronic stress and immune*) and located some 86 in the 47,607 abstract records^21^. Supplementary Table 4 in the Supplementary Materials lists those found, together with their categories of action. Table 4 breaks out frequencies of occurrence pre–COVID-19 (i.e., through 2019) vs. in COVID-19 times (i.e., 2020 publication dates). For instance, of the 3,290 records pertaining to vaccines, 2,415 have publication dates pre-2020; 875 are dated 2020. So the ratio of 2020 to pre-2020 is only 0.36.^22^

**Table 4:** Shifting frequency of attention to various approaches regarding COVID-19.

**Records**	**Categories**	**1949–2019** **(18,451)**	**2020** **(29,156)**	**Ratio** **(1.58)**
3,290	Vaccination	2,415	875	0.36
2,120	Restrict exposure	351	1,769	5.04
1,183	Antiviral medications	211	972	4.61
1,028	Biotoxins	935	93	0.10
992	Iatrogenic	368	624	1.70
801	Lifestyle	256	545	2.13
159	Occupational/environmental	80	79	0.99
6	Psychological/social/economic	3	3	1.00

Patterns of occurrence are intriguing. Note that the 1949–2019 dataset reflects 39% of the total 47,607 records; the 2020 data, 61%, albeit concentrated in a very short span of some 6 months. The ratio of 2020/pre-2020 articles is 1.58. That said, some points of interest are as follows:

➢ Vaccine focus in 2020 is relatively less than previously.
➢ Focus on the other two tactical (virological) categories is way up.
➢ Focus on the strategic (toxicological) categories is relatively steady.

These tabulations suggest that the major focus of 2020 research has been on virological approaches under the duress of the pandemic. Attention to both restricting exposure and antivirals is up about 5-fold. Further, we know that attention to vaccine development is high, but the nature of that in the compressed 2020 timeframe, with proprietary and clinical trialing considerations, may suppress its consideration in the open research literature. One might imagine considerable attention being directed to vaccine safety, but a search of the 47,607 abstract records spots only 36 mentioning that phrase.

On the other hand, attention to the more strategic approaches oriented to bolstering immune systems is not absent, but neither is it up proportionately.

### Topic Evolutionary Pathways

Zhang et al. (2017) have developed an approach of SEPs to track the evolution of topical emphases over time (In the original algorithm, they define a topic as a group of similar articles and geometrically represent it as a circle, with a centroid measured by the mean of all involved articles, and a boundary measured by the largest Euclidean distance between the centroid and all other articles). The algorithm then categorizes articles published in one time period (e.g., a year), and thus, the entire dataset is analyzed as a bibliometric stream. Along the stream, SEP processes articles one by one—assigning each article to its most similar topic, and based on the Euclidean distance between an article and the centroid of its assigned topic, deciding whether this article belongs to the exact topic (i.e., the distance is within this topic's boundary) or a sub-topic (i.e., the distance is larger than the boundary, indicating a potential drift of knowledge occurs). Then, the relationship between the topic and its sub-topics is defined as a predecessor–descendant relationship—the key to understanding the evolution of topics. Besides, the algorithm labels a topic as the term sharing the highest average similarity with all other involved terms, but if such a term has already been used for labeling, it will use the second one, and so forth.

We configured the bibliometric stream with the following strategy, and in total, we set 26-time slices for the COVID-19 case:

1) We grouped articles published between 1949 and 1980 as the initial topic of the evolutionary pathways and label as “coronavirus.”
2) The following two time slices respectively cover articles from 1981 to 1990, and from 1991 to 2002, since a rapid increase in the number of articles occurred in 2003 due to the SARS pandemic.
3) From 2003 to 2019, each year is set as a time slice; and
4) We divided the data of 2020 into months, and thus, six time slices are obtained.

**Figure 4:**
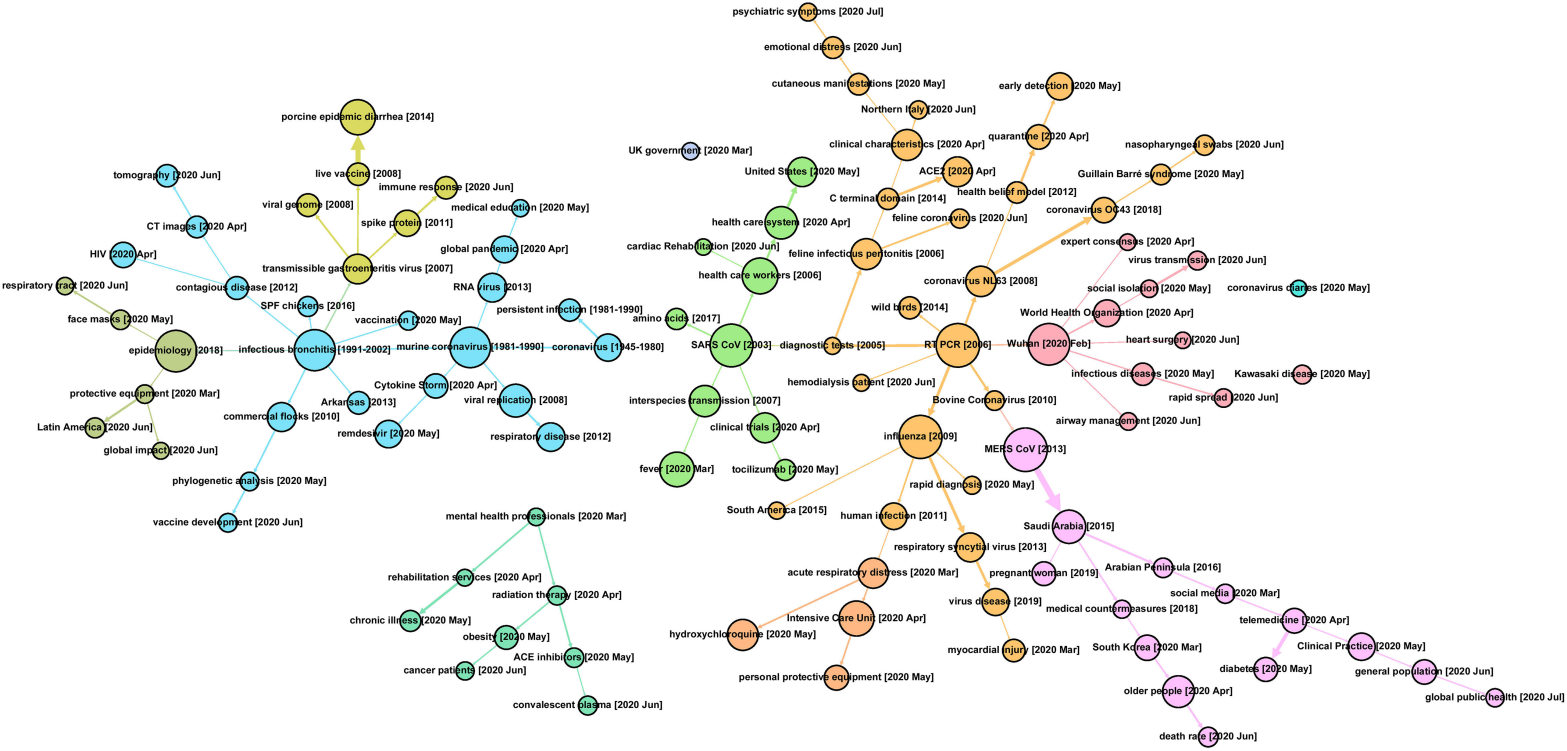
Topic Evolution Pathways in COVID-19 Research [based on analyses of the 47,607 core PubMed search as of July 1, 2020].

Figure 4 presents a complex developmental profile for the 47,607 COVID-19–related research articles, which could help gain perspective on topical heritage in at least four ways.^23^ For one, a reader can locate a topic of recent interest and observe its associations with prior research topics. To illustrate, toward the center–right edge, we note a recent emphasis in June 2020, on “rapid spread.” That draws upon work on infectious diseases, in turn nucleated by studies about Wuhan (February 2020).

A second approach is to step back and note some 10 color-coded concentrations of research, which is generated by the approach of community detection integrated into Gephi as “modularity” (Newman, 2006). Large nodes (node size indicates the number of studies) that appear to nucleate various ongoing research threads include (moving from left to right in Figure 4) epidemiology, infectious bronchitis, murine coronavirus, SARS CoV, reverse transcriptase–polymerase chain reaction (RT-PCR), Wuhan, influenza, and MERS CoV.

Third, digging into these research threads can help locate one's own, or others' research emphases, possibly suggesting information resources worth mining. One can then examine related research threads of particular interest. Some could include the following:

➢ Early coronavirus research conducted before 2003 seems mostly related to murine coronavirus. After 2003, topics within this thread establish the foundation of coronavirus research, especially in molecular virology, such as the viral genome, spike protein, and RNA virus. Thus, after the COVID-19 pandemic, new paths, including clinical characteristics (e.g., immune response and Cytokine Strom), testing approaches (e.g., CT images), and vaccine development, might uncover certain key knowledge bases of testing, treating, and preventing COVID-19.
➢ SARS studies, from 2003, as the year of the SARS pandemic—the first human coronavirus outbreak recorded after the Millennium—provide roots extending out several paths to certain common features of COVID-19, such as interspecies transmission and fever. Two highlights are observed along with this thread: concerns in the healthcare system (maybe especially for the United States) during April and May 2020, and tocilizumab, a drug considered for COVID-19 treatment.
➢ RT-PCR (from 2006) displays half a dozen or so threads, including diagnostic testing links to SARS, branches for different types of coronaviruses, and strong association with influenza research. It is not surprising that at the early stage of the COVID-19 pandemic, compared to virologic research, public health issues, closely linked with protection, control measures, testing, and early detection of COVID-19 are raised by, not only the general public but also academia. Besides that, topics such as emotional distress and psychiatric symptoms, together with Northern Italy, are observed in June 2020 and thereafter.
➢ MERS (2013) ties closely to Saudi studies and South Korea, highlighting the second human coronavirus outbreak after SARS, and along pathways involving different demographic groups. Current concerns could well link back to the experience of handling the MERS crisis, like social media and telemedicine.
➢ Wuhan (2020, February) marks the starting locale of the COVID-19 outbreak, and the size of this node also indicates the incredible number of articles published in such a short time. Specifically, this thread tracks to WHO roles and a range of clinical and public health concerns.

A fourth perspective looks at the degree of connectivity of particular topics in terms of network analytics. For example, toward the bottom left of Figure 4, note a separate, small tree structure emanating from mental health professional concerns. Or in the upper central region, note a “UK government” node not connected to other topics at the threshold of the figure. Examination of the SEP can suggest opportunities for new connections to bolster the transfer of research knowledge.

Topic connectedness is itself of interest. We calculated the similarity (degree of relation) among the 109 topics in the map (Figure 4). Prior to 2020 (i.e., prior to COVID-19 emergence), this domain showed a level of similarity of 0.12. In contrast, 2020 topics show a lower similarity of 0.03 with topics generated prior to 2020. Furthermore, the average similarity among active 2020 topics is 0.03 as well. This suggests a relatively weakly connected research domain. This could reflect the “explosion” of truly novel research or a hurried research environment wherein full review of the extant knowledge is not taking place.

### The Research Community

This section explores some aspects of the “players” active in producing these 47,607 journal publications.^24^ It gingerly addresses research questions:

➢ How well-connected are the players?
➢ Can we gauge the disciplines contributing?
➢ Does COVID-19 constitute a singular research community?

**Figure 5:**
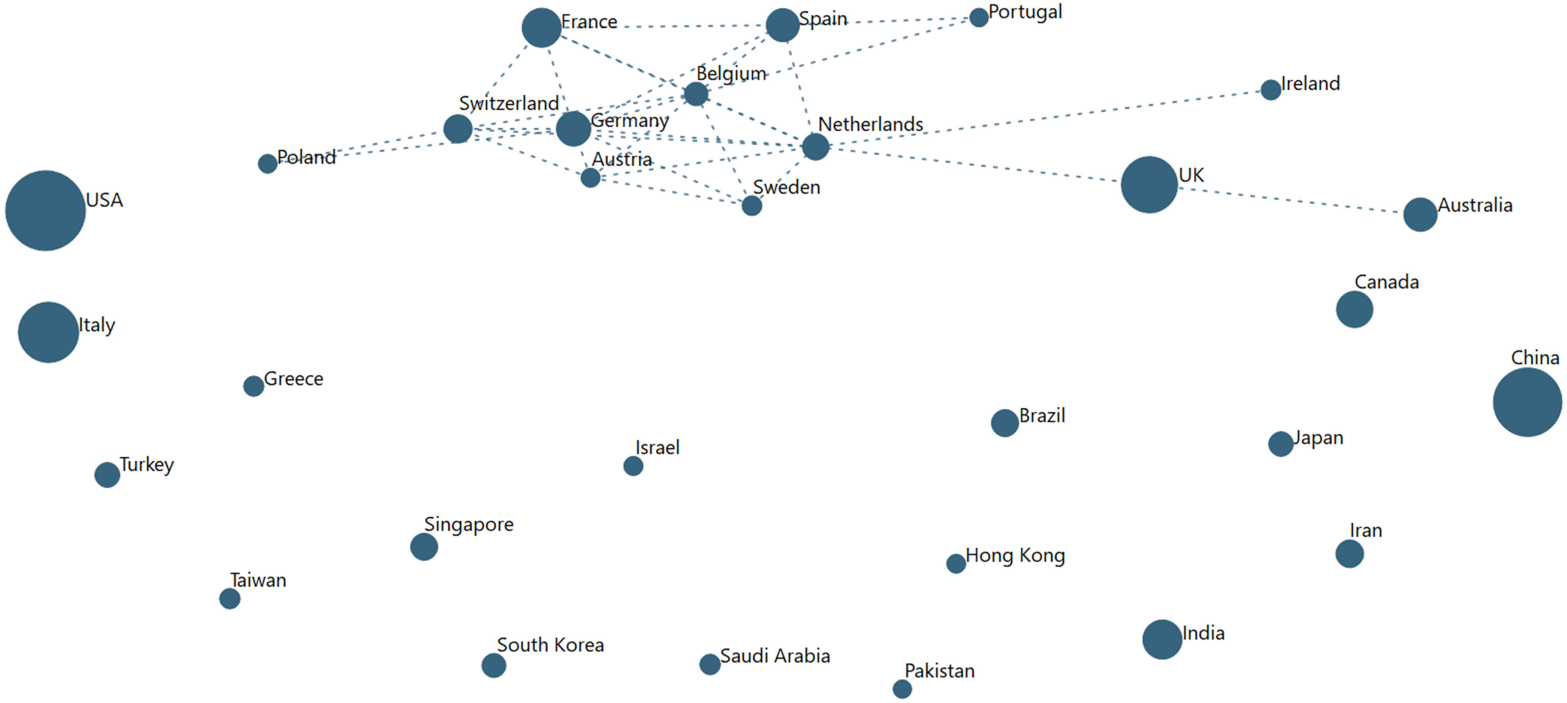
The Extent of Co-authoring among the Top 30 Countries (for COVID-19 papers in 2020). Node size reflects number of papers published, 1949-2020 (to date, July 2); links indicate substantial degree of co-authoring, shown above a threshold.

As mentioned, we see author affiliations in a host of countries—some 191. To get a sense of the extent of international collaboration, we mapped international co-authorship in various ways. Possibly most informative is a look at the top 30 countries for 2020 publications (each representing >200 articles co-authored, as of July 1−29,156 of the 47,607 records). Each has more than 200 articles in the set. Figure 5 shows a *VantagePoint* autocorrelation map (country with country). The strongest connection is among European countries. An especially richly interconnected core network showing a high proportion of articles co-authored with four or more other nations includes the Netherlands, Germany, Belgium, Switzerland, Spain, Austria, France, and Sweden.

Of note, the leading two contributors have been preponderantly working alone (not internationally collaborating) this year:

➢ USA—of 6,101 articles in 2020, 532 with China; 507, the UK, 441, Italy; 339, Canada
➢ China—of 4,418 articles in 2020, 532 with the USA; 246, UK; 145, Australia; 142, Italy^25^

A map of the top 20 (each over 100 articles) pre-2020 (18,451 records) is not drastically different. A core European group collaborates extensively, including the UK. Also connecting are Saudi Arabia and Egypt. The two leaders show as quite independent then as well.

Fry et al. (2020) found increased USA–China collaboration from COVID-19–related research pre-2020 (3.6% of global articles together) to 2020 research (4.9% co-authored together). Our data just above find such USA–China collaboration up from 1.1 to 1.8% of global publication totals.

We leave investigation at the organization or individual level to individuals who could use the website to identify connections of particular players of interest.

Another community question concerns disciplinary engagement and interactions. The most direct indicator of such information from PubMed records is the MeSH Qualifiers. We explore the extent to which particular of the 2020 records (29,156) are indexed into various pairs of Qualifiers. We limit to the most prevalent Qualifiers for this year's COVID-19 data. These 19 Qualifiers are associated with between 530 (psychology) and 4,310 (epidemiology) records.^26^ On average, these records show 4.2 of the 19 top Qualifiers. To the extent that these indicate multidisciplinary:

➢ Most multidisciplinary Qualifiers are pathogenicity (5.0) and isolation and purification (4.9).
➢ Least multidisciplinary are psychology (3.3) and epidemiology (3.4).

**Figure 6:**
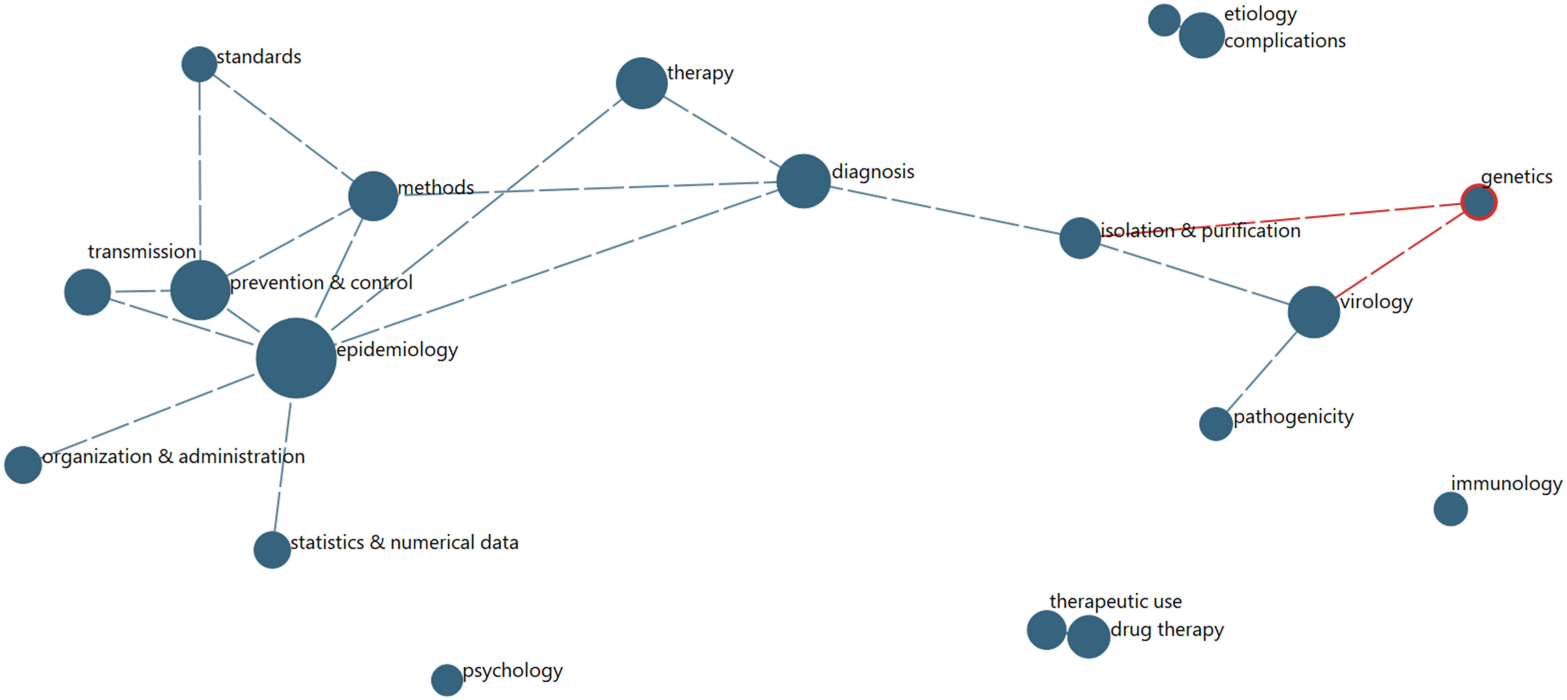
MeSH Qualifiers Multidisciplinary Network (for 2020 Records). Node size reflects number of papers associated with the given frequent MeSH Qualifer; links indicate substantial degree of co-occurrence, shown above a threshold.

Figure 6 shows the degree of affinity among the top 19 Qualifiers (those associated with 500 or more articles) for the 29,156 articles with 2020 dates. Arguably, this shows two clusters^27^:

➢ Epidemiology centers a network of seven other Qualifiers that tend to be associated with it (as records are indexed by National Library of Medicine indexers for MEDLINE).
➢ Virology centers a network of 3 other Qualifiers.

## Discussion

### Profiling COVID-19 Research

The research enterprise is stressed by the upsurge in research outputs—Figure 1 and Supplementary Figure 3 offer two perspectives on the hyper-growth in PubMed-indexed core COVID-19 journal articles. That is accentuated by other modes of sharing research results, particularly via preprints (e.g., bioRxiv, medRxiv, arXiv). Park (2020) notes the overload on the peer review system caused by the flood of articles on COVID-19.

Brainard (2020) points out the stress on researchers trying to keep up with pertinent literature; the outpouring is too excessive for traditional monitoring and reading approaches to suffice. This provides an opportunity for research profiling, such as this, to aid researchers and research managers in identifying valuable research knowledge.

We provide high-level tabulations to overview the research domain—Who? (Table 1); Where? (international collaboration, Figure 5); When? (Figure 1 and Supplementary Figure 3); and What (e.g., Table 3)? We offer select combinations of those aspects—e.g., multidimensional (Figure 3 and Supplementary Figure 2); and What/When? (Table 4 and Figure 4). The intent is to orient readers to pertinent research knowledge, that they might pursue in more detail via provided web resources (https://searchtechnology.github.io/VPDashboard/; http://www.techminingforglobalgood.org/open-covid-19-research-for-analysis/).

We have manipulated “What?” information—i.e., *topical content*—in several ways, seeking to depict research patterns and priorities. These start with the rich MeSH indexing of PubMed content (c.f., Table 2 and Figure 6 concerning MeSH Qualifier distribution across leading countries and Qualifier interconnections). We present ways to compile MeSH Primary Term topical content across MeSH Levels (Figure 2 and Supplementary Tables 1–3). We apply Scientific Evolution Pathways (Figure 4) to identify topics and their interconnections, showing the evolution over time periods. Furthermore, Table 3 reports on emerging COVID-19 topics, whereas Supplementary Table 4 tabulates topical activity categorized into eight tactical or strategic categories of action to counter COVID-19. So, we offer multiple, and we hope, complementary, means to elicit topical intelligence.

It would seem desirable that future research give stronger attention to the strategic, immune-boosting factors (Table 4). Recognizing that viral exposures cannot be generally eliminated, strengthening immune reactions offers greater promise to mitigate future infections (Chandra, 1996; Davison et al., 2016; Kostoff et al., 2020a,b; Tsatsakis et al., 2020). In Kostoff's view, increased attention to factors that diminish immune system capability is sorely needed. Study of their roles and ways to reduce their effects on the populace should be directed at

➢ lifestyle factors (e.g., factors contributing to obesity, smoking, alcohol and substance abuse)
➢ occupational/environmental factors (e.g., air pollution, radiation exposures, chemical exposures, and even deforestation—Dobson et al., 2020).

### Opening LBD Opportunities

This article overviews our COVID-19 research profiling. As noted, a keen aim in so doing is to inform LBD. We actually intend to pursue discovery processes not limited to just finding never-before investigated factors that could contribute to mitigating COVID-19 or future viral threats. We want to combine research knowledge accrued in COVID-19–related research with additional knowledge resident in other literature. Kostoff (Kostoff, 2011, 2019; Kostoff and Patel, 2014) labels this “literature-related discovery and innovation,” but we will use LBD as shorthand.

Our COVID-19 topical analyses (see section Discussion) seek to provide multiple means to get at essential and evolving topics. They do not, in themselves, provide a menu of missing research knowledge to guide LBD. We need a COVID-19 knowledge model to structure efforts to resolve critical elements to mitigate the pandemic and in turn point to needs that LBD might serve. We see good potential in the use of the SEPs (Figure 4) as a starting point, with the MeSH indexing helpful in organizing knowledge resources. Compared to scientometric reviews on COVID-19 (Colavizza et al., 2020; Kagan et al., 2020; Zhang L. et al., 2020), our analyses, particularly with the engagement of the SEPs, highlight the discovery of COVID-19's historical knowledge flows. This treats the full knowledge base, which then become a distinctive feature of our work—that is, discovering objective insights from the literature in a data-driven and semiautomatic approach, with limited human intervention.

Preliminary results offer potential bases for future LBD analyses. Consolidating topical entities from the current COVID-19 abstracts and phrases is complemented by extraction of MeSH terms in several categories. For instance, several diseases (e.g., diabetes, hypertension, cardiovascular diseases, anxiety) appear in the recent (2020) segment of our data, whereas they were rarely mentioned in the pre-2020 related coronavirus research. Contrast between COVID-19 research (2020) and prior coronavirus research indicate emerging drugs (e.g., hydroxycholoroquine, ribavirin, remdesivir, lopinavir). Combinations provide another potential stepping stone (e.g., lopinavir and ritonavir). Also, certain genes show elevated attention in the recent COVID-19 literature (e.g., S surface glycoprotein [severe acute respiratory syndrome coronavirus 2]; ORF1ab polyprotein [Severe acute respiratory syndrome coronavirus 2]; and N nucleocapsid phosphoprotein). Future LBD probes could be started by scanning for topics linked to such diseases, drugs, or genes associated with COVID-19 in other (non–COVID-19) literatures. We plan to explore within PubMed and beyond (e.g., WoS).

High on our future research agenda is to build on these empirical topic summarizations to engage subject matter experts in identifying critical gaps in understanding COVID-19 and devising effective remedies. Table 4 arrays eight categories pertaining to tactical and strategic approaches to counter COVID-19 and future viral threats. Those categories comprise a number of components (Supplementary Table 4), some of which could warrant LBD ventures to introduce remote research knowledge to enrich COVID-19 research focuses. We will seek expert synthesis and extension of our empirical topical information to identify problematic virus attributes, critical biomechanisms, and biomarkers and treatment approaches potentially suitable for “repurposing” (Kostoff, 2019, 2020).

Application of artificial intelligence tools holds special promise in organizing and exploring this research explosion. Brainard (2020) notes more than 1,500 data science projects to use the CORD-19 information resources to address COVID-19 issues. We intend to direct *VantagePoint* machine learning capabilities to help sort the COVID-19 topical content into the categories just noted (c.f., MeSH categories, emergent topics, tactical/strategic approaches). These can complement expert knowledge by reducing the labor entailed—i.e., based on modest trial datasets to auto-categorize more extensive COVID-19 and out-of-domain record sets.

Once we have identified a small number of high-potential topical elements, we will pursue complementary information resources. We would start with PubMed (beyond COVID-19), ClinicalTrials.gov, and Web of Science (to extend basic science coverage). We are open to other sources as well.

## Data Availability Statement

The details of our search strategy, target databases, and data pre-processing can be found in the article/Supplementary Material. This data can also be found here: http://www.techminingforglobalgood.org/open-covid-19-research-for-analysis/. Core COVID-19 dataset of 47,607 abstract records via an interactive web viewer at https://searchtechnology.github.io/VPDashboard/.

## Author Contributions

AP coordinated the analyses and led the paper writing. YZ and MW primarily provided the scientific evolutionary pathway (SEP) for topical analyses. YH primarily provided the multi-level MeSH analyses. All authors contributed to the article and approved the submitted version.

## Conflict of Interest

AP was employed by the company, Search Technology, Inc.

The remaining authors declare that the research was conducted in the absence of any commercial or financial relationships that could be construed as a potential conflict of interest.

## Footnotes

^1^See the website: http://arrowsmith.psych.uic.edu/arrowsmith_uic/index.html.

^2^Colavizza et al. (2020) analyzed CORD-19 (46,996 records), comparing to Web of Science (WoS) and identifying interesting “macrotopics.”

^3^Since June, the interface limits downloads to the first 10,000 records, so we ran a combination of partial searches. We did so using separate searches this time, for select year sets (the interface gives a slider to choose years) and complementary “AND/NOT” searches. For instance, one query added “AND clinical” to the core query and downloaded resulting records; that was followed by another query adding “NOT clinical” to get record sets within the 10,000 limit. We recombine the downloaded sets in *VantagePoint* on the desktop, remove duplicates, perform essential data cleaning and consolidation, and then post on our indicated website.

^4^In MEDLINE detail: “COVID-19”[All Fields] OR (“coronavirus”[MeSH Terms] OR “coronavirus”[All Fields]) OR “corona virus”[All Fields] OR “2019-nCoV”[All Fields] OR “SARS-CoV”[All Fields] OR “MERS-CoV”[All Fields] OR “severe acute respiratory syndrome”[All Fields] OR “Middle East respiratory syndrome”[All Fields].

^5^We use the legacy, peer-reviewed journal articles for consistency from the “PubMed” interface option. The “PMC” option has more content, including preprints, many lacking metadata indexing and some full text that does not combine well with analyses of abstract records.

^6^For some of our analyses, we approximate month of publication. The PubMed “date published” field contains various details—e.g., “2020 June,” “2020,” “2020 June 1,” “2020 Summer,” “2020 Sep–Oct,” etc. Furthermore, publication dates are uncertain with different policies on dating digital and print versions, journal issue “pre-dating,” etc. So our dating is approximate.

^7^We calculate emergence using 10 time periods from September 2019, through June 2020.

^8^PubMed distinguishes 62 types of publications in the dataset, led by journal articles (76%).

^9^To address the challenges of PubMed affiliation variations, the *VantagePoint* List Cleanup (using “Extract main”) script incorporates regular expressions (regex) and particular fuzzy matching routines.

^10^The *VantagePoint* NLP is based on the Princeton WordNet approach for Part of Speech (PoS) tagging, but its noun phrase extraction differs as it is tailored to keep scientific and technical terms intact (e.g., chemical formulas).

^11^*VantagePoint* software, and the related *Derwent Data Analyzer* from Clarivate, focuses primarily on aiding analyses of research publication and patent abstract record compilations. It has been applied in many studies, particularly in conjunction with “tech mining” (Porter and Cunningham, 2005). Explorations of its algorithms, applications, and assessments can be found in conjunction with the annual Global Tech Mining (GTM) conferences—https://vpinstitute.org/global-techmining-conference/global-techmining-conference-2020/—and in some hundred or so papers on the VPI website—https://vpinstitute.org/.

^12^Compiled over years from multiple resources and empirical results (e.g., by running a list cleanup routine and saving matches to a thesaurus).

^13^Affiliation information in PubMed is somewhat varied. These values reflect matching of items, but not in full detail. We combine units (e.g., hospitals) with their primary organizations (e.g., universities).

^14^See the links: https://www.researchgate.net/project/COVID-19-Research-Literature-Profiling-and-Mining; and http://www.techminingforglobalgood.org/open-covid-19-research-for-analysis/.

^15^These are approximate given the vagaries of publication dating. In our download on July 1, we have 2,503 publications dated beyond June. For these analyses, we include them in “June on.”

^16^See the website: http://www.iarpa.gov/index.php/research-programs/fuse.

^17^We refined the resulting 1,380 Emergent Terms to 1,347 by culling 33 very general terms appearing in many records (e.g., COVID-19), locations (e.g., United Kingdom), or less interesting terms (e.g., illness).

^18^Leaving out a few with minimal substantive interest—e.g., “# of patients” —and consolidating two variants of ACE2.

^19^These are all “emergent” topics based on strong growth in attention over the months from September 2019 through June, 2020. Table 2 shifts this comparison extending back in time from 1949 onward.

^20^https://www.researchgate.net/project/COVID-19-Research-Literature-Profiling-and-Mining.

^21^Some of the terms, particularly some associated with lifestyle (e.g., substance abuse) and psychological/social/economic (e.g., aspects of stress), may appear in COVID-19-related research literature as causal factors diminishing immune system health, but also as effects of the pandemic and its treatment. This prompts consideration of specifying contingencies to separate the immune health aspects that are of most interest here.

^22^We compare differences for this set of publications; we do not apply statistical significance testing to these differences to infer to other datasets.

^23^Note that in Figure 4 each node represents a research topic, and an arrow represents the pre-decessor-descendant relationship between its connected nodes, and the thickness of an arrow then indicates the strength of such a relationship.

^24^We note that the data are not completely cleaned. PubMed formatting of player information can be challenging. We developed an improved List Cleanup aid— “organization names, extract main” —and thesauri to consolidate variations of the same organization for the COVID-19 data; these helped considerably.

^25^Here, Hong Kong is treated separately.

^26^The MeSH Qualifiers are not complete for these data. They are available for only 52% of the 47,607 records. Further, they are present for only 27% of the 2020 publications here. So results are only suggestive of disciplinary interactions.

^27^The nodes reflect number of records; the links reflect relative degree of co-occurrence; the map could be drawn to show many more links at a lower threshold. Further, note the limitation of only partial coverage of the record set discussed in the previous footnote.
