# Supplementary material for: Tracking and Mining the COVID-19 Research Literature
Source: Front Res Metr Anal. 2020 Nov 6;5:594060. doi: 10.3389/frma.2020.594060 (PMC8025982; doi:10.3389/frma.2020.594060)
Supplement: Supplementary file 1 [file Data_Sheet_1.docx]

Supplementary Material

# Supplementary Figures and Tables

## Supplementary Figures


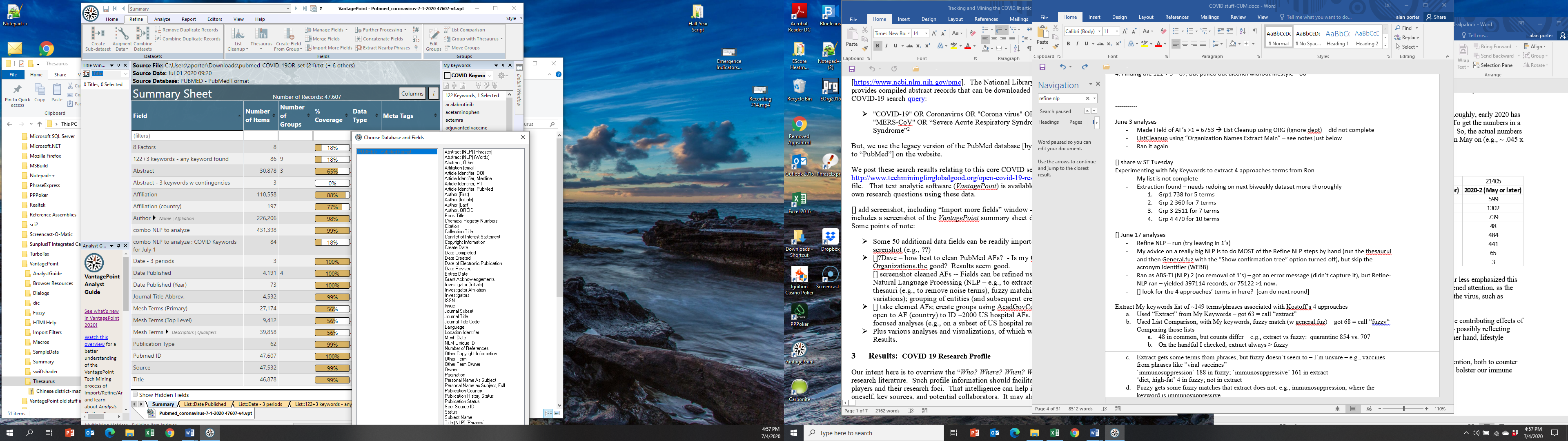


**Supplementary Figure 1.** VantagePoint Text Analysis Software Summary Sheet [also showing Window for Importing Additional Fields]


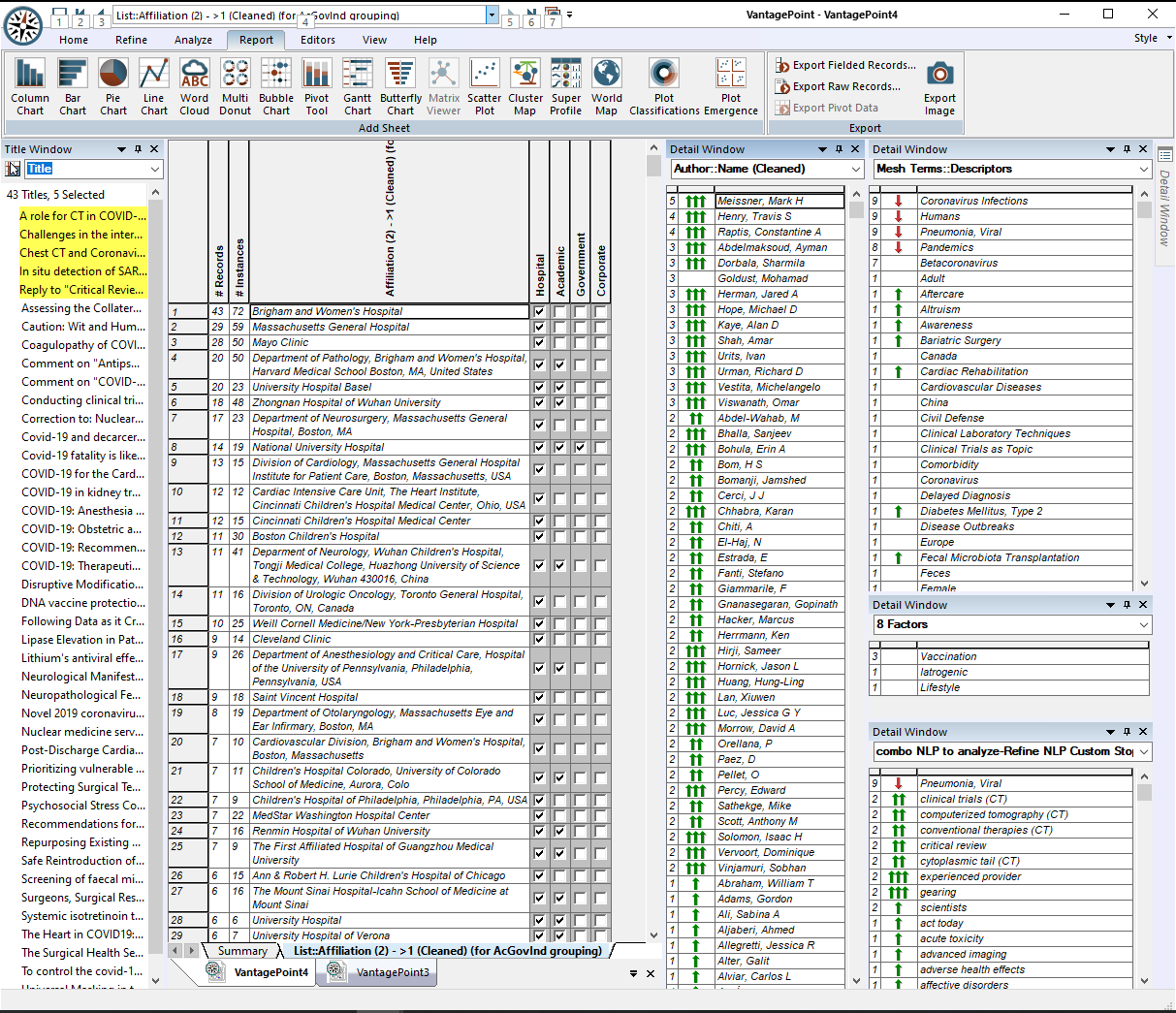


**Supplementary Figure 2.** Screenshot of a “Zoom-in” on Papers Authored by Brigham and Women’s Hospital in 2020 [with detail windows open to show authors and topics]


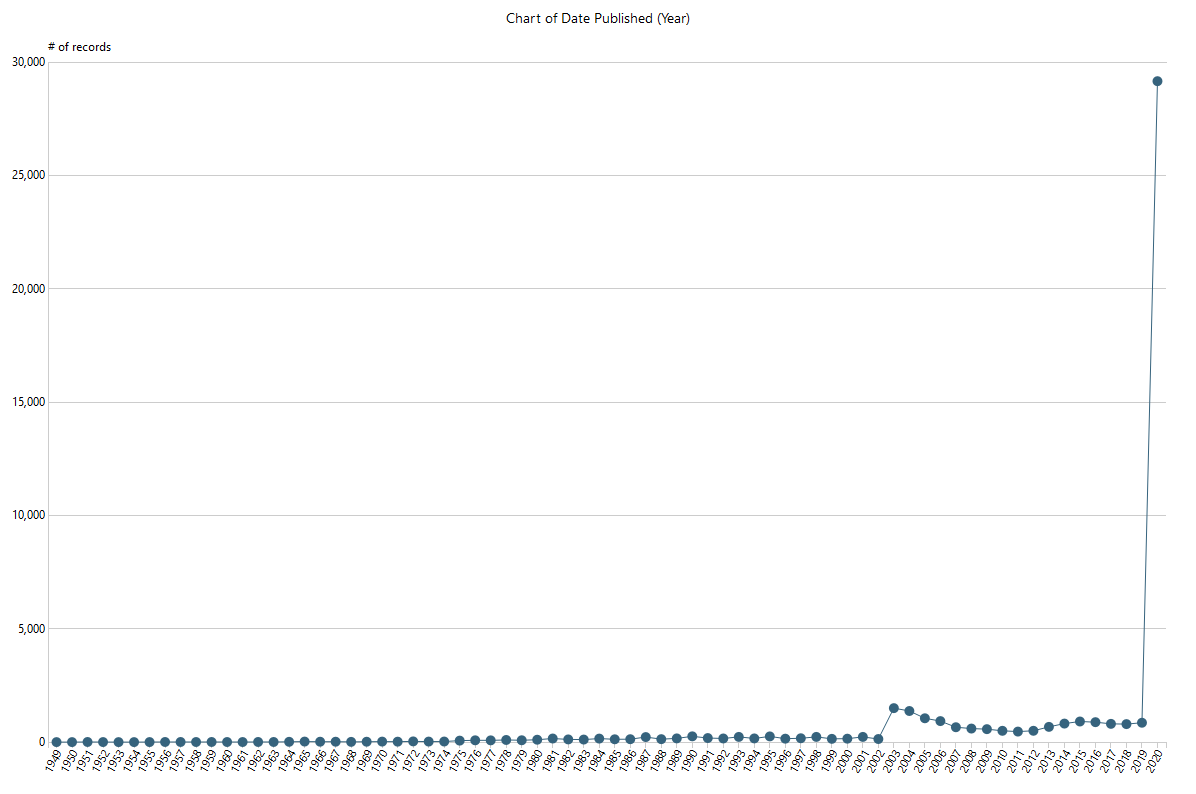


**Supplementary Figure 3.** The Trend in COVID-19-related Research: 1949-2020 (part-year)

## Supplementary Tables

**Supplementary Table 1.** Top Primary Level 2 MeSH Terms (for the 47,607 PubMed Records)

| NO | Mesh Terms (Primary) (Level 2) | Records | NO | Mesh Terms (Primary) (Level 2) | Records |
| --- | --- | --- | --- | --- | --- |
| 1 | Infections | 19361 | 11 | Eukaryota | 1902 |
| 2 | Viruses | 16068 | 12 | Biological Factors | 1787 |
| 3 | Respiratory Tract Diseases | 13375 | 13 | Chemical Actions and Uses | 1622 |
| 4 | Environment and Public Health | 7164 | 14 | Health Care Quality, Access, and Evaluation | 1551 |
| 5 | Amino Acids, Peptides, and Proteins | 5726 | 15 | Therapeutics | 1526 |
| 6 | Animal Diseases | 3745 | 16 | Enzymes and Coenzymes | 1415 |
| 7 | Investigative Techniques | 3446 | 17 | Cells | 1381 |
| 8 | Health Care Facilities, Manpower, and Services | 2294 | 18 | Health Occupations | 1361 |
| 9 | Pathological Conditions, Signs and Symptoms | 2274 | 19 | Health Services Administration | 1351 |
| 10 | Genetic Phenomena | 2029 | 20 | Nucleic Acids, Nucleotides, and Nucleosides | 1304 |

**Supplementary Table 2.** Top Primary Level 3 MeSH Terms (for the 47,607 PubMed Records)

| NO | Mesh Terms (Primary) (Level 3) | Records | NO | Mesh Terms (Primary) (Level 3) | Records |
| --- | --- | --- | --- | --- | --- |
| 1 | Virus Diseases | 18473 | 11 | Enzymes | 1407 |
| 2 | RNA Viruses | 15642 | 12 | Diagnostic Techniques and Procedures | 1224 |
| 3 | Respiratory Tract Infections | 13145 | 13 | Health Services | 1205 |
| 4 | Lung Diseases | 8539 | 14 | Biological Products | 1203 |
| 5 | Public Health | 6999 | 15 | Medicine | 1203 |
| 6 | Proteins | 5588 | 16 | Nucleic Acids | 1104 |
| 7 | Animals | 1768 | 17 | Peptides | 1084 |
| 8 | Hepatitis Viruses | 1720 | 18 | Antigens | 1017 |
| 9 | Pathologic Processes | 1607 | 19 | Virus Physiological Phenomena | 1016 |
| 10 | Pharmacologic Actions | 1524 | 20 | Glycoconjugates | 991 |

**Supplementary Table 3.** Top Primary Level 4 MeSH Terms (for the 47,607 PubMed Records)

| NO | Mesh Terms (Primary) (Level 4) | Records | NO | Mesh Terms (Primary) (Level 4) | Records |
| --- | --- | --- | --- | --- | --- |
| 1 | RNA Virus Infections | 17297 | 11 | Chordata | 1654 |
| 2 | Positive-Strand RNA Viruses | 15148 | 12 | Blood Proteins | 1481 |
| 3 | Pneumonia | 8203 | 13 | Globulins | 1356 |
| 4 | Pneumonia, Viral | 8079 | 14 | Disease Attributes | 1238 |
| 5 | Disease Outbreaks | 5177 | 15 | Glycoproteins | 1169 |
| 6 | Severe Acute Respiratory Syndrome | 3858 | 16 | Therapeutic Uses | 1109 |
| 7 | Viral Proteins | 3047 | 17 | Vaccines | 1024 |
| 8 | Membrane Proteins | 1938 | 18 | Hydrolases | 1023 |
| 9 | Public Health Practice | 1747 | 19 | Epidemiologic Methods | 991 |
| 10 | Murine hepatitis virus | 1674 | 20 | RNA | 982 |

**Supplementary Table 4.** Terms Associated with Particular Categories of Action re: The Pandemic

| Particular Categories of Action | Terms |
| --- | --- |
| Restrict Exposure | facemask (56); hand wash (76); hand-washing (6); large gathering (7); lockdown (721); quarantine (1104); sanitize (32); social distanc (143); social isolation (227) |
| Tactical | acalabrutinib (1); Anakinra (18); apremilast (5); arbidol (30); Avigan (1); azithromycin (182); baricitinib (17); Canakinumab (2); colchicine (31); convalescent plasma (167); EIDD-2801 (3); favipiravir (57); Fc receptor (16); fingolimod (10); Galidesivir (4); hydrocholoroquine (1); ilaris (2); ivermectin (26); Kaletra (12); leronlimab (1); Lopinavir (286); methylprednisolone (132); nanomedic (12); oseltamivir (76); remdesivir (215); ruxolitinib (17); sarilumab (12); scorpion venom (2); Tamiflu (3); Tocilizumab (165); tofacitinib (3); umifenovir (16) |
| Vaccination | virus interference (2); vaccin (3249); antibody-dependent (73) |
| Lifestyle | substance abuse (11); smoking (149); obes (621); i-rev (33); high-fat diet (2); alcohol AND lifestyle (10) |
| Iatrogenic | serotonin reuptake inhibitor (1); NSAID (29); immunosuppr (507); iatrogenic (12); gamma radiation (1); antiretroviral (48); antibiotic (334); anesthetic (47); anaesthetic (19); adjuvanted vaccine (9); acetaminophen (12) |
| Biotoxins | T-2 toxin (1); rhinovirus (700); respiratory syncytial virus (759); Pseudomonas aeruginosa (13); mycotoxin (2); Aflatoxin (2) |
| Occup/Env | WiFi (1); UV radiation (5); ultraviolet radiation (7); sodium fluoride (2); pesticide (7); perfluorooctanoic acid (1); PAHs (2); nanoparticle (89); Microplastics (1); insecticide (5); herbicide (1); heavy metal (2); fine particulate (4); endocrine disrupt (1); crude oil (2) |
| Psych/Soc/Econ | stressful life events (2); chronic stress AND immun (4); childhood adversity (1) |
